# Supplementary material for: Human Cortical Pyramidal Neurons: From Spines to Spikes via Models
Source: Front Cell Neurosci. 2018 Jun 29;12:181. doi: 10.3389/fncel.2018.00181 (PMC6034553; doi:10.3389/fncel.2018.00181)
Supplement: Supplementary file 1 [file Data_Sheet_1.docx]

Supplementary Material

**Human cortical pyramidal neurons:**

**From spines to spikes via models**

**Guy Eyal, Matthijs** **B. Verhoog, Guilherme Testa-Silva, Yair Deitcher, Ruth Benavides-Piccione, Javier DeFelipe, Christiaan P.J. de Kock, Huibert D. Mansvelder and Idan Segev**

*** Correspondence:**
Idan Segev
idan@lobster.ls.huji.ac.il

# Supplementary Figures and Tables

## Supplementary Figures


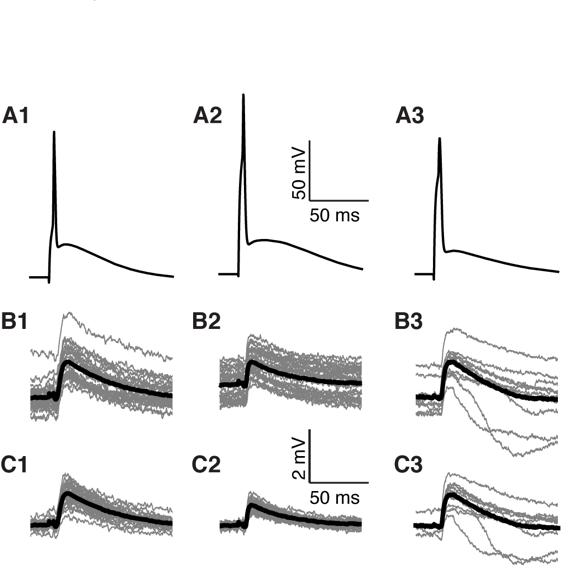


**Figure S1: Paired recordings from synaptically connected human L2/L3 PCs.** (**A1**) A spike (30 repetitions) was initiated in a presynaptic HL2/L3 PC. (**B1**) The population EPSP’s (grey traces) in response to the presynaptic spike shown in (**A1**); the average EPSP is depicted by the dark line. (**C1**) As in B1 when the based-lines of all EPSPs are merged; the average (dark line) was used as the target EPSP for the model fits in **Figure 1**. The EPSP shown here corresponds to the EPSP underlying the “pink fit” in **Figure 1** (110426_Sl4_Cl2_4to6 in **Table S1**). (**A2-C2**) Similar to (**A1-C1**), but for an additional HL2/L3 PC pair (Blue fit in **Figure 1**, 110426_Sl4_Cl2_6to4 in **Table S1**). (**A3-C3**) Another pair (yellow fit in **Figure 1**, 110322_Sl2_Cl2_6to4 in **Table S1**); in this case 10 presynaptic spike repetitions were initiated experimentally. The average traces for ten connected HL2/L3 PCs pairs (**Table S1**) are available in the modelDB (<http://modeldb.yale.edu/238347>).


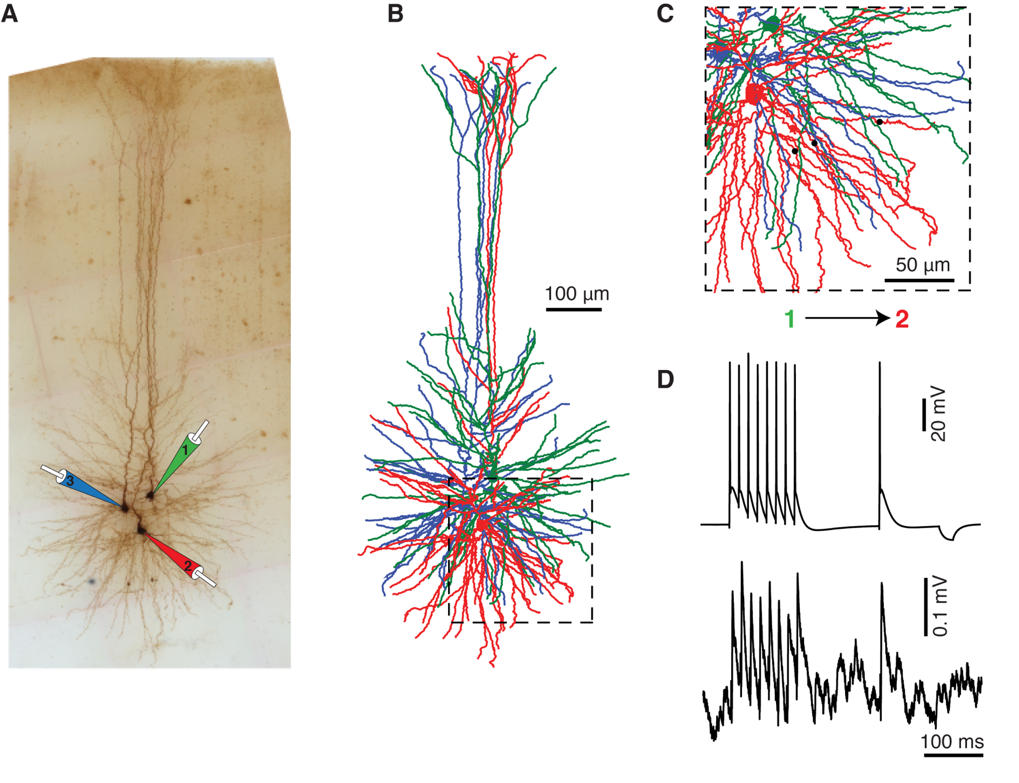


**Figure S2. Location of putative synapses between connected HL2/L3 PCs pairs. (A)** Slice from a fresh human tissue from human temporal cortex with three stained HL2/L3 PCs. Triplet somatic recording was performed in these cells. **(B)** 3D reconstruction of the cells in **(A)** (see also (Mohan et al., 2015)). **(C)** Zoom in to the basal region marked by a square in **(B).** Location of three putative synapses (black dots) between axon of cell #1 (green cell, the axon is not shown) and dendrites of cell #2 (red). Note that the synapses are on the basal tree about 100 μm from the soma (as predicted by our model in Figure 1). **(D)** Functional synapse between cell #1 (top frame – presynaptic spikes) and cell #2 (lower frame - resultant EPSPs). No functional connection was found between cell #2 (blue cell) and cell #1.


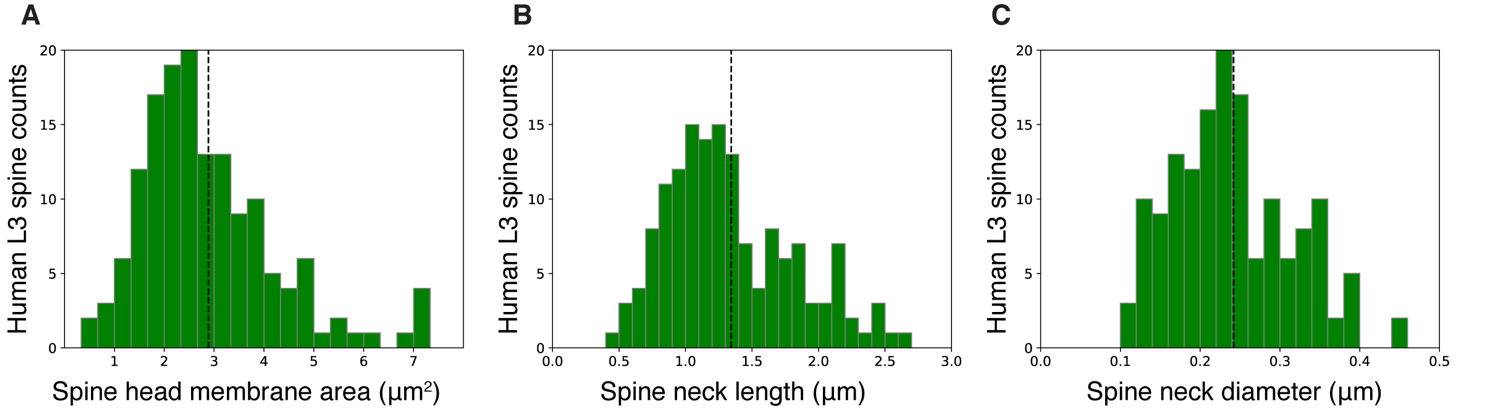


**Figure S3. Dimensions of human L3 spines.** Histograms of spine head membrane area (**A**), spine neck length (**B**), and spine neck diameter (**C**), values for the 150 human L3 spines analyzed. Dashed black line represents the mean of all spines.

**
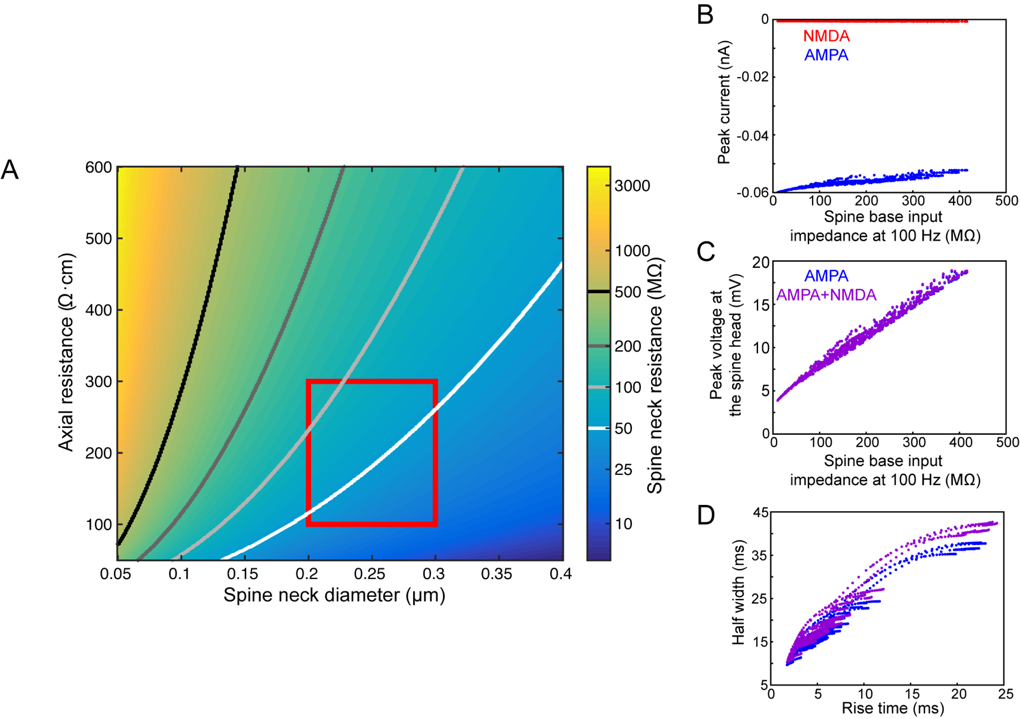
**

**Figure S4. Modeling human dendritic spines. (A)** The dimensions of the human dendritic spine imply low spine neck resistance values**.** Spine neck resistance values as a function of the spine neck diameter and the axial resistance in the spine neck. The red square depicts the likely regime for the spine neck resistance (19 MΩ – 128 MΩ), with spine neck diameter ranging from 0.2 µm to 0.3 µm and the specific neck resistance from 100 Ωcm to 300 Ωcm. White, bright grey, dark gray, and black lines represent spine neck resistance values of 50, 100, 200, 500 MΩ, respectively. **(B-D)**, Activation of an excitatory spinous synapse (with AMPA- and NMDA- conductances properties as in Figure 4) generates an EPSP that is carried mainly by AMPA current. **(B)** The peak AMPA (blue) and NMDA (red) currents underlying activation of one spinous synapse as a function of the input impedance at the spine base (measured at input frequency of 100 Hz). **(C)** As in **(B)** but for the peak EPSP in the spine head, with (purple) and without (blue) the NMDA conductance. Note the linear relationship which results from the fact that the main frequency underlying the AMPA kinetics is ~100 Hz. **(D)** The shape index curve at the soma for synapses with (purple) and without (blue) NMDA- conductance.


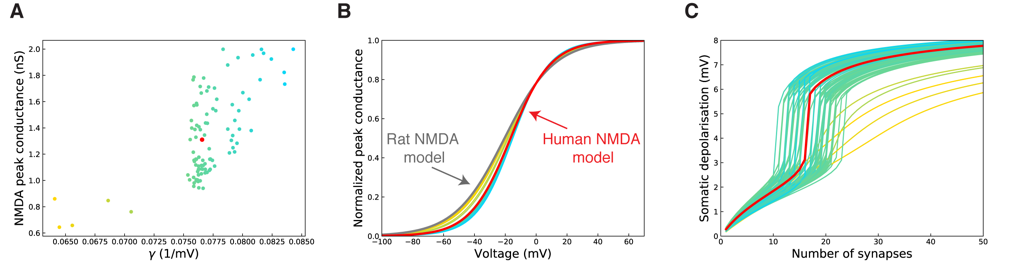


**Figure S5**. **Exhaustive parameter search to fit human NMDA data.** Results of 100 best fits to the experimental EPSPs shown in **Figure 4B**. **(A)** Distribution of NMDA conductance and *γ* (the NMDA “steepness” parameter in **Eq. (6)**) for the best 100 models. Red point depicts the best typical model (shown in Figure 4B and used throughout this study). Dots are color coded according to their *γ* value. **(B)** Dependence of NMDA peak conductance on voltage. Note that, for all models, NMDA conductance near the resting potential (-86 mV) is close to zero. Gray, rat NMDA models as in (Jahr and Stevens, 1990) and (Sarid et al., 2013). Note that in human, the dependence of the NMDA-conductance on voltage is steeper. **(C)** Somatic voltage response as a function of the number of AMPA + NMDA synapses activated on a single basal dendrite shown in Figure 5F. Colors in (**B**) and (**C**) are as in (**A**).

**
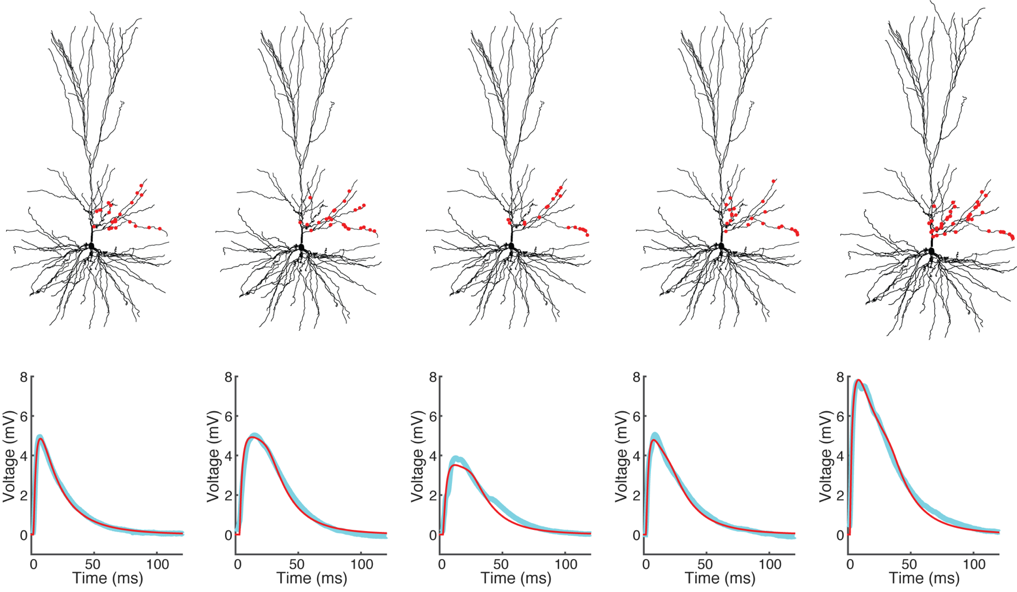
**

**Figure S6. AMPA + NMDA model fits composite somatic EPSPs in additional five experiments.** Top, as in Figure 4, but for five additional experiments done in three HL2/L3 pyramidal cells (light blue traces at bottom, see **Methods**). The synaptic model for AMPA and NMDA components was that shown in Figure 4, and only the number of synapses and their dendritic locations were changed to obtain an optimal fit (minimal RMSD) between experimental and model transients (red traces). The corresponding synaptic locations are depicted by the red dots superimposed on the dendritic tree.

**
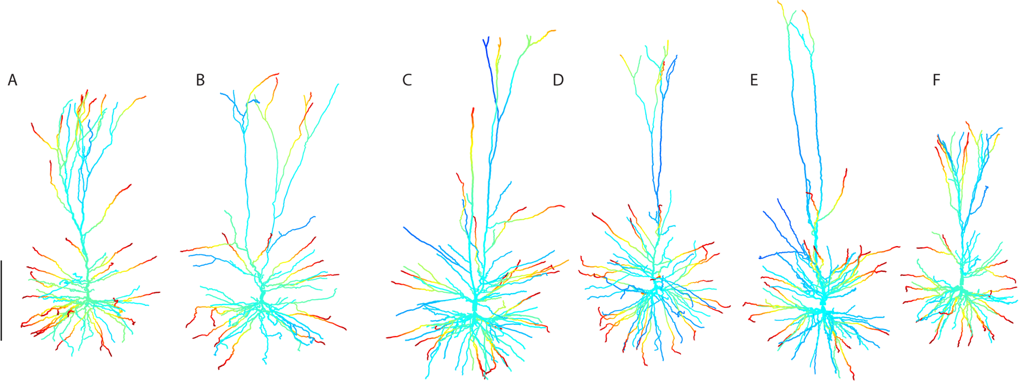
**

**Figure S7. Human dendrites support multiple simultaneous independent NMDA-spikes.** The maximal number of NMDA spikes that could be generated simultaneously and independently in the six modeled human L2/L3 PCs. **(A)** (blue model in Figure 7): 32 NMDA spikes; **(B)** (green model in Figure 7): 18 NMDA spikes; **(C)** (red model in Figure 7): 25 NMDA spikes; **(D)** (pink model in Figure 7): 22 NMDA spikes; **(E)** (orange model in Figure 7): 24 NMDA spikes, **(F)** (cyan model in Figure 7): 28 NMDA spikes. Scale bar 300 $\mu$m.

**
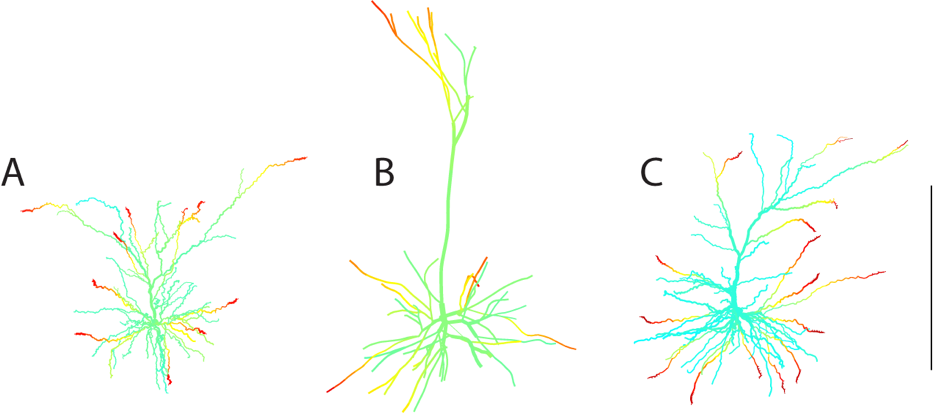
**

**Fig. S8. Multiple NMDA-based functional subunits in rat L2/3 pyramidal cells.** The maximal number of NMDA spikes that could be generated simultaneously and independently in three modeled rat L2/3 PCs. Compare to **Figure 6** and **Figure S7.** (**A**) Sarid et al., 2013, model 110602A: 11 NMDA spikes; (**B**) Markram et al. 2015: 14 NMDA spikes; (**C**) Sarid et al., 2013, model 280503A: 16 NMDA spikes. Scale bar 300 $\mu$m, note the difference in size from the HL2/L3 PCs in **Figure S7**.

**
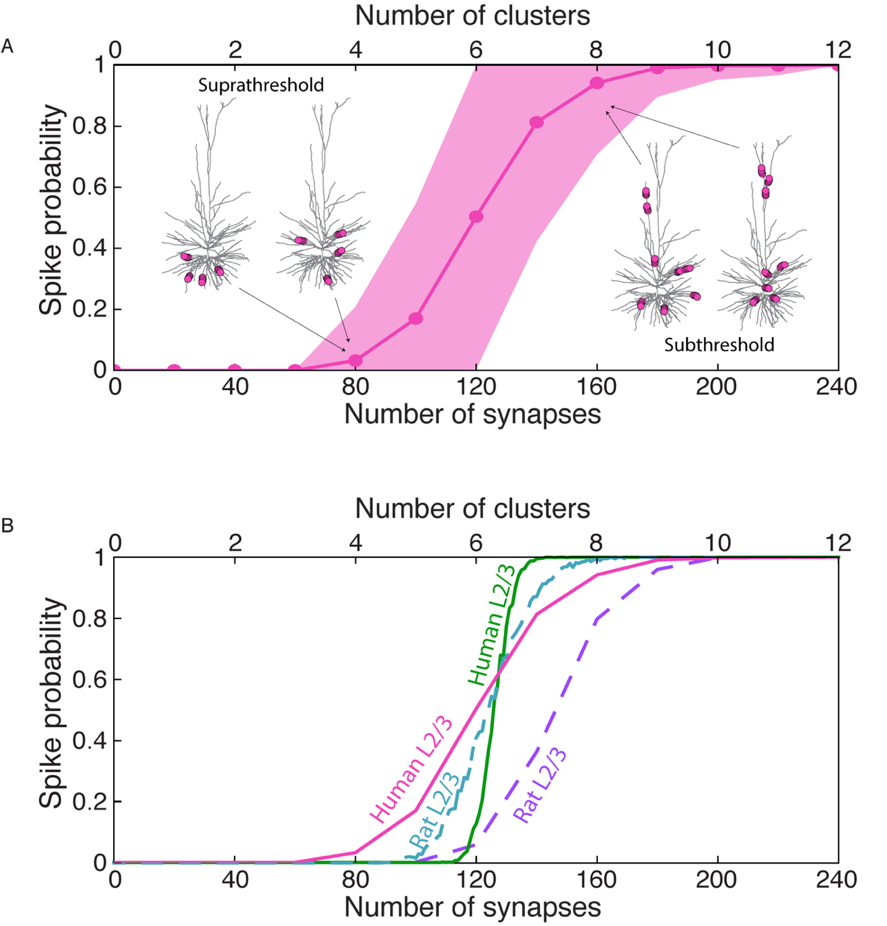
**

**Figure S9**. **(A)** The dendritic locations of clustered synaptic inputs strongly affects the number of clusters required for the activation of somatic Na^+^ spike. The pink curve describes the spike probability as a function of the number of activated clusters (same as in Figure 8A). On the left, two cases are shown where four proximal clusters (20 synapses each) were sufficient to generate a somatic spike. Right, two examples whereby eight synaptic clusters do not generate a somatic AP. For the case of eight clusters, in the left, some of the clusters are located on the same branch, or on a near-by branch (in electrotonic terms) and, thus, they sum sublinearly (voltage saturation), while in the right-most case, many of the clusters are located on dendritic branches with low input resistance, such that 20 synapses are insufficient for generating local NMDA spikes. **(B)** Number of excitatory synapses required to generate a somatic Na^+^ spike in human and rat models. Pink and green curves represent the clustered and distributed cases, respectively, for the HL2/L3 PC model in **A** and in Figure 8. Purple and cyan dashed curves represent the clustered and distributed cases, respectively, for L2/3 PC rat model (see **Methods**).

# Supplementary tables

**Table S1. Properties of AMPA-conductance in human L2/L3 – L2/L3 PCs synapses.** EPSPs from ten connected HL2/L3 PCs pairs were recorded (pair name is shown at the left column). Model fit to experimental EPSPs (as in Figure 1**D**) provided the peak synaptic conductance values when either a single synaptic contact was assumed (middle column) or five contacts per connection were assumed (right column). Locations of modeled synapses were derived as explained in Figure 1 and in the **Methods**. Equations **(2-5)** describe the synaptic model used for fitting experimental and modeled EPSPs.

| **Pair Name** | **Peak synaptic conductance assuming one contact (nS)** | **Peak synaptic conductance assuming 5 contacts (nS)** |
| --- | --- | --- |
| 081212_1to5 | 6.99 ± 2.29 | 0.79 ± 0.07 |
| 081212_2to3 | 1.47 ± 0.05 | 0.28 ± 0.05 |
| 081212_5to1 | 1.84 ± 0.07 | 0.36 ± 0.16 |
| 100316_Cl2_4to3 | 7.75 ±0.13 | 1.54 ± 0.01 |
| 100316_Cl4_5to2 | 2.53 ± 0.22 | 0.47 ± 0.01 |
| 110322_Cl3_Cl1_1to5 | 6.47 ± 0.50 | 1.21 ± 0.03 |
| 110322_Cl3_Cl1_1to6 | 14.04 ± 1.01 | 2.61 ± 0.07 |
| 110322_Sl2_Cl2_6to4 | 3.24 ± 0.58 | 0.51 ± 0.01 |
| 110426_Sl4_Cl2_4to6 | 4.33 ± 0.48 | 0.70 ± 0.02 |
| 110426_Sl4_Cl2_6to4 | 1.51 ± 0.06 | 0.29 ± 0.01 |
| **average** | **5.02 ± 3.74** | **0.88 ± 0.70** |

**Table S2. Properties of NMDA-conductance in human L2/L3 PCs.** Fitting experimental EPSPs following extracellular stimulation (see **Figure 4**) to a synaptic model that included AMPA- and NMDA- components (**Eqs. 2-6)**. The best 100 models were accepted, providing the optimal location, number of synapses and parameters for the NMDA- and AMPA- conductances. Parameters for the best typical model were used in **Figures 4 - 8** are shown in the lower row.

|  | **Number of activated Synapses** | **NMDA peak conductance (nS)** | $\boldsymbol{\tau}_{\boldsymbol{rise}}$ **NMDA (ms)** | $\boldsymbol{\tau}_{\boldsymbol{decay}}$**NMDA (ms)** | **n (1/mM)** | **γ (1/mV)** | **AMPA peak conductance (nS)** | **Mean distance of synapses from soma (µm)** |
| --- | --- | --- | --- | --- | --- | --- | --- | --- |
| **100 best models** | **23.5±3.9** | **1.32±0.32** | **7.1±1.3** | **35.4±0.8** | **0.28** | **0.077± 0.003** | **0.63 ± 0.13** | **182±9** |
| **Model in Figure 4C** | **21** | **1.31** | **8.0** | **35.0** | **0.28** | **0.077** | **0.73** | **195** |

**Table S3. Mean and SD values of the spiking features of L2/L3 human pyramidal cells used for the MOO algorithm.** The first twelve features were extracted from a minimum of 10 spike train repetitions obtained following a 1 sec long suprathreshold step depolarizing current to the PCs modeled in this study.
The current input amplitudes that resulted with these spike trains were 700 pA for cell 060303; 700 pA for cell 060308; 700 pA for cell 060311; 550 pA for cell 130303; 550 pA for cell 130305 and 600 pA for cell 130306. The last five features were taken from the normalized I-F curve of 25 other HL2/L3 PCs **(see Methods).**

| **Features** | **Cell 060303 (blue)** | **Cell 060308 (green)** | **Cell 060311 (red)** | **Cell 130303 (orange)** | **Cell 130305 (cyan)** | **Cell 130306 (pink)** |
| --- | --- | --- | --- | --- | --- | --- |
| **Voltage base (mV)** | -86.82 ± 0.53 | -83.99 ± 0.20 | -83.32 ± 1.44 | -78.77 ± 0.65 | -85.93 ± 0.41 | -82.17 ± 0.26 |
| **Steady state voltage (mV)** | -88.07 ± 0.58 | -87.29 ± 0.31 | -86.76 ± 1.37 | -80.36 ± 0.79 | -87.89 ± 0.33 | -85.19 ± 0.39 |
| **Mean frequency (Hz)** | 10.38 ± 0.31 | 9.17 ± 0.39 | 10.00 ± 1.15 | 9.25 ± 0.27 | 10.67 ± 0.29 | 8.24 ± 0.30 |
| **Time to first spike (ms)** | 21.65 ± 0.74 | 17.98 ± 0.23 | 19.93 ± 2.54 | 30.00 ± 1.24 | 40.16 ± 2.22 | 31.60 ± 0.94 |
| **Burst ISI (ms)** | 10.30 ± 1.16 | 8.73 ± 0.29 | 7.69 ± 0.24 | 27.19 ± 2.25 | 40.71 ± 3.13 | 14.67 ± 1.03 |
| **ISI CV** | 0.0680 ± 0.0210 | 0.1533 ± 0.0481 | 0.4509 ± 0.1068 | 0.1038 ± 0.0253 | 0.0593 ± 0.0080 | 0.0514 ± 0.0118 |
| **Adaptation index** | -0.0088 ± 0.0059 | -0.0194 ± 0.0040 | 0.0963 ± 0.0436 | -0.0205 ± 0.0067 | -0.0089 ± 0.0070 | -0.0070 ± 0.0056 |
| **AP height (mV)** | 41.35 ± 0.20 | 35.92 ± 0.41 | 35.52 ± 0.93 | 39.12 ± 0.35 | 36.24 ± 0.38 | 43.20 ± 0.20 |
| **AP begin voltage(mV)** | -58.27 ± 0.53 | -48.64 ± 0.33 | -57.05 ± 0.76 | -53.00 ± 0.52 | -51.70 ± 0.18 | -55.09 ± 0.18 |
| **AHP depth abs (mV)** | -70.33 ± 0.46 | -67.58 ± 0.62 | -66.53 ± 0.77 | -64.87 ± 0.54 | -67.25 ± 0.16 | -67.88 ± 0.43 |
| **AHP time from peak (mV)** | 3.04 ± 0.05 | 3.22 ± 0.76 | 4.11 ± 0.25 | 2.71 ± 0.69 | 3.16 ± 0.07 | 3.25 ± 0.10 |
| **Spike half width (ms)** | 1.08 ± 0.02 | 1.01 ± 0.02 | 1.22 ± 0.05 | 0.76 ± 0.02 | 1.08 ± 0.02 | 0.98 ± 0.02 |
| **Mean frequency 0.75 (Hz)** | 3.43 ± 2.03 | 2.67 ± 1.61 | 3.22 ± 1.95 | 2.70 ± 1.63 | 3.63 ± 2.15 | 2.01 ± 1.24 |
| **Mean frequency 1.25 (Hz)** | 16.59 ± 2.83 | 15.29 ± 2.73 | 16.22 ± 2.82 | 15.37 ± 2.79 | 16.92 ± 2.86 | 14.14 ± 3.07 |
| **Mean frequency 1.5 (Hz)** | 21.70 ± 4.84 | 20.28 ± 4.91 | 21.34 ± 4.89 | 20.35 ± 4.93 | 22.04 ± 4.84 | 19.20 ± 5.34 |
| **Mean frequency 2.0 (Hz)** | 31.31 ± 7.81 | 30.20 ± 7.94 | 31.02 ± 7.85 | 30.28 ± 7.94 | 31.64 ± 7.83 | 27.74 ± 9.47 |
| **Mean frequency 3.0 (Hz)** | 42.20 ± 2.79 | 43.53 ± 7.04 | 41.62 ± 2.90 | 43.67 ± 7.05 | 42.90 ± 2.87 | 37.43 ± 12.17 |

**Table S**4**. Properties of ten membrane ion channels and related parameters that fit the somatic spiking activity in HL2/L3 PCs.** These parameters were extracted via multiple objective optimization procedure (see **Methods**); models for each parameter in this Table is as described in (Hay et al., 2011).

| **Model 130306 (pink)** | **Model 130305 (cyan)** | **Model 130303 (orange)** | **Model 060311 (red)** | **Model 060308 (green)** | **model 060303 (blue)** | **Parameter** | **Sections** |
| --- | --- | --- | --- | --- | --- | --- | --- |
| -8.19E+01 | -8.58E+01 | -8.01E+01 | -8.31E+01 | -8.38E+01 | -8.12E+01 | e_pas (mV) | all |
| 4.65E+00 | 3.08E+00 | 5.23E+00 | 6.00E+00 | 5.53E+00 | 4.94E+00 | gNaTgbar_NaTg (S/cm2) | axonal |
| 5.70E+00 | 4.01E+00 | 6.11E+00 | 8.61E+00 | 4.43E+00 | 1.50E+01 | slopem_NaTg | axonal |
| 9.20E+00 | 1.00E+01 | 1.57E+01 | 8.44E+00 | 1.32E+01 | 8.11E+00 | vshiftm_NaTg (mV) | axonal |
| 5.55E+00 | 6.06E-02 | 9.87E+00 | 1.20E+01 | 1.19E+01 | 1.02E-02 | vshifth_NaTg (mV) | axonal |
| 3.02E-05 | 6.84E-02 | 7.34E-04 | 7.09E-04 | 6.40E-03 | 1.26E-02 | gSK_E2bar_SK_E2 (S/cm2) | axonal |
| 1.99E+00 | 2.00E+00 | 1.75E+00 | 1.72E+00 | 1.98E+00 | 1.83E+00 | gSKv3_1bar_SKv3_1 (S/cm2) | axonal |
| 8.61E-06 | 4.77E-04 | 8.05E-04 | 2.95E-04 | 7.67E-05 | 9.99E-04 | gCa_LVAstbar_Ca_LVAst (S/cm2) | axonal |
| 5.00E-04 | 7.09E-05 | 9.30E-04 | 7.84E-04 | 7.18E-04 | 9.85E-04 | gCabar_Ca (S/cm2) | axonal |
| 1.63E-03 | 9.12E-04 | 8.76E-03 | 3.32E-02 | 1.06E-02 | 4.29E-02 | gamma_CaDynamics_E2 | axonal |
| 2.01E+01 | 9.90E+01 | 2.09E+02 | 5.87E+02 | 3.00E+02 | 4.66E+02 | decay_CaDynamics_E2 (ms) | axonal |
| 2.83E-02 | 2.05E-02 | 4.13E-02 | 4.99E-02 | 1.46E-04 | 6.71E-05 | gNap_Et2bar_Nap_Et2 (S/cm2) | axonal |
| 3.01E-03 | 9.91E-02 | 3.20E-03 | 1.23E-04 | 7.11E-03 | 1.97E-03 | gK_Pstbar_K_Pst (S/cm2) | axonal |
| 2.48E-02 | 9.97E-02 | 9.46E-02 | 9.53E-02 | 1.04E-03 | 5.61E-04 | gK_Tstbar_K_Tst (S/cm2) | axonal |
| 8.55E-04 | 5.43E-04 | 5.11E-06 | 1.44E-05 | 8.05E-04 | 8.83E-04 | gImbar_Im (S/cm2) | axonal |
| 3.83E-01 | 2.98E-01 | 6.39E-01 | 1.69E-01 | 3.30E-01 | 1.74E-01 | gNaTgbar_NaTg (S/cm2) | somatic |
| 9.27E+00 | 1.46E+01 | 1.24E+01 | 1.38E+01 | 9.83E+00 | 1.39E+01 | slopem_NaTg | somatic |
| 8.00E+00 | 1.60E+01 | 1.60E+01 | 1.60E+01 | 7.67E+00 | 8.00E+00 | vshiftm_NaTg (mV) | somatic |
| 7.02E+00 | 1.16E+01 | 2.14E-02 | 1.19E+01 | 1.19E+01 | 1.10E+01 | vshifth_NaTg (mV) | somatic |
| 9.66E-02 | 9.82E-02 | 1.15E-02 | 9.99E-02 | 3.11E-02 | 9.14E-02 | gSK_E2bar_SK_E2 (S/cm2) | somatic |
| 5.02E-02 | 5.19E-02 | 7.20E-04 | 7.12E-02 | 1.32E-01 | 9.29E-02 | gSKv3_1bar_SKv3_1 (S/cm2) | somatic |
| 1.00E-03 | 6.60E-04 | 9.95E-04 | 9.29E-04 | 5.19E-04 | 9.97E-04 | gCa_LVAstbar_Ca_LVAst (S/cm2) | somatic |
| 1.75E-05 | 4.74E-07 | 5.60E-07 | 6.17E-04 | 5.22E-05 | 7.03E-04 | gCabar_Ca (S/cm2) | somatic |
| 5.99E-03 | 1.46E-02 | 1.41E-02 | 5.28E-04 | 7.98E-03 | 7.63E-04 | gamma_CaDynamics_E2 | somatic |
| 3.05E+02 | 4.31E+02 | 3.85E+02 | 2.38E+02 | 1.97E+02 | 1.64E+02 | decay_CaDynamics_E2 (ms) | somatic |
| 2.26E-04 | 4.76E-05 | 6.76E-05 | 5.72E-05 | 3.56E-05 | 2.14E-06 | gNap_Et2bar_Nap_Et2 (S/cm2) | somatic |
| 8.37E-05 | 1.18E-03 | 7.42E-05 | 1.82E-05 | 1.94E-04 | 3.66E-07 | gK_Pstbar_K_Pst (S/cm2) | somatic |
| 8.99E-02 | 7.92E-02 | 1.59E-03 | 7.57E-02 | 9.10E-02 | 4.80E-02 | gK_Tstbar_K_Tst (S/cm2) | somatic |
| 4.11E-04 | 9.83E-04 | 3.07E-05 | 1.21E-04 | 1.10E-05 | 2.63E-04 | gImbar_Im (S/cm2) | somatic |
